# A potential new biological marker of the biological age

**DOI:** 10.3389/fgene.2013.00198

**Published:** 2013-10-24

**Authors:** Victor A. Zuev, Marina V. Mezentseva, Galina M. Schaposchnikova

**Affiliations:** Department of Medical Microbiology, Gamaleya Institute for Epidemiology and Microbiology, Ministry of Health of Russian FederationMoscow, Russia

**Keywords:** biological markers, biological age, neurons, gliosis, age factors

For many years, data have been accumulated which point to a significant similarity between the nature of the brain damage in the aging process (in the case of dementia or even in outwardly healthy elderly people) and the brain damage caused by prion diseases of humans and animals (Sobel, 1970; Fraser and McBride, 1985; Masters et al., 1985; Gajdusek, 1988). In both cases, lesions are characterized by the two fundamental processes—the loss of neurons and an active proliferation of glial cells—gliosis (Finch, 2002; Kim and de Vellis, 2005; Deng et al., 2006; Bessis et al., 2007).

In case of prion diseases, neuronal cell death was originally coupled with the action of an infectious agent, while gliosis was always considered only as a reparative response of glial cells to the destruction of neurons (Brown, 1997; Ironside and Bell, 1997; Finch, 2002). However, an infectious agent is absent in the aging process, so the reason for the primary neuronal cell death is also absent. These arguments led us to a basic hypothesis about the primary role of gliosis in the development of the aging process, which, as we supposed, results in the death of neurons. We assumed that the distribution of glial elements in the confined space could easily disturb the nutrition of the neuron (Zuev, 2001). Indeed, the interaction between a neuron and a brain capillary is not direct and is realized through an intermediary, the astrocyte, rather than through its (neuron) close proximity to the wall of the capillary (Magistretti et al., 1999). It is also known that this liaison is not sufficiently strong (Cotrina and Nedergaard, 2002). Therefore, all of this allows us to suppose that an active reproduction of the surrounding glial cells may be quite sufficient to break this fragile relationship between an astrocyte and a neuron, on the one hand, and an astrocyte and a capillary, on the other hand, which will eventually lead a neuronal cell to “starve to death.”

It is possible that in order to trigger the process of active cell proliferation in the course of aging, a specific factor (“the aging factor”), which will stimulate this active proliferation of glial cells, needs to be accumulated in the brain tissue. In order to find this factor of aging, we conducted the experiments described below.

The first series of experiments were aimed at determining the stimulating effect of the brain extracts obtained from young and old mice on the proliferation of glial cells in primary dissociated and transplantable cultures. Extracts of the brain from young mice had a weak (less than 2-fold) stimulatory effect on the proliferation of glial cells. In contrast, the brain extracts obtained from aged mice had a pronounced 3 to 4-fold stimulatory effect (Zuev et al., 2000). The factor which stimulates the proliferation of glial cells in culture was detected in the brain tissue of mice starting from 10 months of age. At the same time, a similar cytoproliferative factor was also found in the blood serum of old mice; however, it was never detectable in the blood serum of young animals (Zuev et al., 2005). Nevertheless, it remained unclear whether the factor of a detectable increase in the proliferation of glial cells was the reason for the neuronal loss or the consequence of this process and, therefore, the process of aging itself. To address this question, we carried out experiments aimed at the artificial aging of young mice. Four groups of animals were studied. Group No. 1 included 10 young 1.5 month-old mice which were daily (10 injections for 2 weeks) intraperitoneally injected with 0.5 ml of a sterile purified brain extract obtained from the young 1.5 month-old mice (first control). Group No. 2 consisted of 10 naturally aged 2-year-old mice (second control). Group No. 3 included 10 young 1.5-month old mice which were daily (10 injections for 2 weeks) intraperitoneally injected with 0.5 ml of a sterile purified brain extract obtained from the aged 2-year-old mice.

Group number 4 consisted of 6 young 1.5-month-old mice which daily (10 injections for 2 weeks) received intraperitoneal injections of 0.5 ml of a blood serum diluted 1: 5, which had been obtained from the aged 2-year-old mice (Table 1). Notably, 3 months after the beginning of the experiment, i.e., when the animals reached 4.5 months of age, the mice from group No. 3 were found to exhibit some clinical manifestations of a premature aging, such as sluggish movements and a slow reaction to food; their hair coat looked dull and less dense with some signs of gray on the ends of hairs. Histological samples of the same regions of the cerebral cortex were prepared after removal of the animals from the experiment. A computer analyzer singled out the total area of neurons on the standard sections of each preparation, with the rest of the area being occupied by the glia (Avtandilov, 2002). As seen from Table 1, the number of neurons proved to be almost 2-fold smaller and gliosis was expressed to a greater extent in the young animals from group No. 3, which had received 10 injections of the brain extract from old mice, as compared with the naturally aged mice from group No. 2. Finally, the findings of this study indicate that the factor stimulating the proliferation of glial cells is detected, in addition to the brain tissue, in the bloodstream of aging mammals. This was supported by the results obtained in the study of the young mice from group No. 4, which received the blood serum from old animals. The mice from group No. 4 also exhibited a remarkable decrease in the number of neurons, as well as the increase in the proliferation of glial cells. Although both of these indicators proved to be less pronounced as compared with those in the mice from group No. 3, they were at the same time more pronounced as compared with the naturally aged 2-year-old mice from group No. 2.

**Table 1:** **Dimension of neuronal and glial parts in the brain-cortex of young, natural, and artificial old mice**.

**Groups**	**Number of cells (n)**	Neuronal areas (in conditional units)^*^	Glial areas (in conditional units)^*^	**Standard deviation ± σ**	**Error of selection ± m**
No 1. Ten young mice which were injected by brain extracts from young mice	419	10.6	89.4	1.15	0.05
No 2. Ten naturally aged 2-years old mice	269	8.4	91.6	1.51	0.08
No 3. Ten young mice which were injected by brain extracts from old mice	190	4.6	95.4	0.42	0.03
No 4. Six young mice which were injected by blood serum from old mice	237	6.3	93.7	0.79	0.05

* - differences between indexes of neuronal areas and glial areas in groups No. 1 and No. 2 are statistical reliable (p < *0.001*; t = 24.4);

- differences between indexes of neuronal areas and glial areas in groups No. 2 and No. 3 are statistical reliable (p < *0.001*; t = 47.50);

- differences between indexes of neuronal areas and glial areas in groups No. 2 and No. 4 are statistical reliable (p < *0.001*; t = 23.3).

These data suggest that a cytoproliferative factor may be present in the brain and blood of aging mice can be regarded as a cause of the aging process, rather than its consequence, which gives grounds to designate it as a “factor of aging.” Detection of the aging factor in the blood serum of mice served as a basis for carrying out preliminary studies in humans. It was found that this factor is detected in human blood serum starting from 25-years of age (Zuev et al., 2005).

Study of the specific properties of the factor have made it possible to reveal that it is resistant to the action of DNase, RNase, trypsin, ultraviolet, ultrasound, and ionizing radiation, but highly sensitive to the effect of proteinase K. The activity of the factor remains constant after heating at 70°C for 30 min; however, the factor undergoes inactivation at 90°C. Its activity is species-specific, i.e., it manifests itself only in the cells of the species, from which the test samples are obtained (Zuev et al., 2005). Based on this, we suppose that the aging factor is a protein of about 10 kDa, which was detected using the Belgian dialysis bags with different pore sizes (50, 25, 15, and 5 kDa) produced by “Orange Scientific” (Belgium) and later confirmed by other researchers (Villeda et al., 2011).

In conclusion, I would like to say a few words about the main concept—the probability of the leading role of gliosis in the aging process of the brain and, eventually, the entire organism. This speculation should not cause surprise for several reasons. Firstly, even in the case of a classical prion infection, such as scrapie, which is characterized, as it would seem, by the primary loss of neurons and subsequent gliosis, a direct analysis of the sequence of events has shown that the activation of microglia and the following cytokine immunoreactivity in the course of the disease occur much earlier than the development of spongiosis. Moreover, it was found that the start of the cytokine production, which is activated by glia, even precedes the process of the neuronal apoptosis (Williams et al., 1996).

Secondly, it has long been demonstrated on the results of experimental studies in mice and rats, as well as based on the data analysis of the autopsy material obtained postmortem from outwardly healthy elderly persons that in the course of a normal aging, different elements of glial cells, particularly astrocytes, are characterized by an increased proliferative activity and cell hypertrophy. At the same time, the authors emphasize that the beginning of the so-called reactive gliosis is characteristic of the early stages of the aging process (Masters et al., 1985; Giese et al., 1998; Cotrina and Nedergaard, 2002; Deng et al., 2006).

As of today we do not have enough evidence knowledge to answer the question what causes or stimulates a cytokine production and more studies should be conducted to establish a definitive nature of these factors.
